# Type I IFN Induction via Poly-ICLC Protects Mice against Cryptococcosis

**DOI:** 10.1371/journal.ppat.1005040

**Published:** 2015-08-07

**Authors:** Edward Sionov, Katrin D. Mayer-Barber, Yun C. Chang, Keith D. Kauffman, Michael A. Eckhaus, Andres M. Salazar, Daniel L. Barber, Kyung J. Kwon-Chung

**Affiliations:** 1 Molecular Microbiology Section, Laboratory of Clinical Infectious Diseases, National Institute of Allergy and Infectious Diseases (NIAID), National Institutes of Health (NIH), Bethesda, Maryland, United States of America; 2 Immunobiology Section, Laboratory of Parasitic Diseases, National Institute of Allergy and Infectious Diseases (NIAID), National Institutes of Health (NIH), Bethesda, Maryland, United States of America; 3 T-Lymphocyte Biology Unit, Laboratory of Parasitic Diseases, National Institute of Allergy and Infectious Diseases (NIAID), National Institutes of Health (NIH), Bethesda, Maryland, United States of America; 4 Division of Veterinary Resources, Office of Research Services, Office of the Director, National Institutes of Health (NIH), Bethesda, Maryland, United States of America; 5 Oncovir, Inc., Washington, DC, United States of America; University of Wisconsin-Madison, UNITED STATES

## Abstract

*Cryptococcus neoformans* is the most common cause of fungal meningoencephalitis in AIDS patients. Depletion of CD4 cells, such as occurs during advanced AIDS, is known to be a critical risk factor for developing cryptococcosis. However, the role of HIV-induced innate inflammation in susceptibility to cryptococcosis has not been evaluated. Thus, we sought to determine the role of Type I IFN induction in host defense against cryptococci by treatment of *C*. *neoformans* (H99) infected mice with poly-ICLC (pICLC), a dsRNA virus mimic. Unexpectedly, pICLC treatment greatly extended survival of infected mice and reduced fungal burdens in the brain. Protection from cryptococcosis by pICLC-induced Type I IFN was mediated by MDA5 rather than TLR3. PICLC treatment induced a large, rapid and sustained influx of neutrophils and Ly6C^high^ monocytes into the lung while suppressing the development of eosinophilia. The pICLC-mediated protection against H99 was CD4 T cell dependent and analysis of CD4 T cell polyfunctionality showed a reduction in IL-5 producing CD4 T cells, marginal increases in Th1 cells and dramatic increases in RORγt+ Th17 cells in pICLC treated mice. Moreover, the protective effect of pICLC against H99 was diminished in IFNγ KO mice and by IL-17A neutralization with blocking mAbs. Furthermore, pICLC treatment also significantly extended survival of *C*. *gattii* infected mice with reduced fungal loads in the lungs. These data demonstrate that induction of type I IFN dramatically improves host resistance against the etiologic agents of cryptococcosis by beneficial alterations in both innate and adaptive immune responses.

## Author Summary

Meningoencephalitis due to *Cryptococcus neoformans* is the leading cause of mortality in AIDS patients in the developing world. It has been known that depletion of CD4 T cells is the most critical predisposing factor to cryptococcosis in HIV infected patients. What has not been clear is the effect of HIV-induced innate inflammation in susceptibility to cryptococcosis. We treated *C*. *neoformans* infected mice with poly-ICLC (pICLC), a dsRNA virus mimic, to study the role of virus-induced type I IFN in host defense against cryptococcosis. PICLC treatment induced type I IFN in *C*. *neoformans* infected mice via MDA5 and significantly prolonged the survival of mice with reduced fungal burden in the brain. PICLC also protected mice from cryptococcosis caused by *C*. *gattii*. PICLC treatment recruited large numbers of neutrophils and Ly6C^high^ monocytes into the lung parenchyma and suppressed eosinophilia. PICLC-mediated protection against *C*. *neoformans* required CD4 T cells and was associated with suppressed Th2 and enhanced Th17 responses. IFNγ and IL-17A were also important for pICLC-induced protection of infected mice. Our study demonstrates that induction of type I IFN dramatically improves host resistance against cryptococci by beneficial alterations in both innate and adaptive immune responses as long as CD4 cells are not depleted.

## Introduction

Cryptococcocal meningoencephalitis is one of the most important AIDS-associated opportunistic infections with an estimated global burden of nearly one million cases with more than 600,000 deaths annually [1]. In fact, the disease is an AIDS defining illness in patients with late-stage HIV infection, particularly in Sub-Saharan Africa and Southeast Asia [1,2]. It is thought that the susceptibility of HIV+ individuals to *C*. *neoformans* infection is primarily due to the depletion of CD4 T cells, which leads to defects in both innate and adaptive immunity [3–5] that predispose to opportunistic infections [6,7]. Indeed, more than 75% of AIDS associated cryptococcosis cases develop in the late-stage of HIV infection when the CD4+ T-lymphocyte count falls below 50 cells/μl [8], and in experimental animal models CD4 T cell deficiency results in defective control of *C*. *neoformans* infection [9–11].

Apart from CD4 T cell depletion, there are many other immunological phenomena that may impact on the ability of the host to control opportunistic infections such as *C*. *neoformans* infection. In particular, large amounts of type I IFNs are produced in response to HIV or SIV infections, which are required to induce potent innate antiviral defense pathways to control viral replication [12–14] and modulate the function of a variety of immune cell types [15,16]. Type I IFN has also been demonstrated to have major effects on the outcomes of bacterial, parasitic and fungal infections [17]. Type I IFN induction, for example, enhanced the susceptibility of mice to infection with *Listeria monocytogenes* [18,19] *Streptococcus pneumoniae* [20] and *Mycobacterium tuberculosis* [21,22] while the opposite was reported for *Mycobacterium avium* infection [23].

Study of this cytokine pathway in fungal infection has been relatively limited, and the contribution of type I IFNs to antifungal immunity has been reported to be either beneficial or detrimental depending on the fungal species [24–28]. In *Candida albicans* infection, type I IFN signaling was reported as required for induction of reactive oxygen intermediates necessary for killing of yeast cells by phagocytic cells [29], while in another study IFN signaling caused no change in fungal burden but resulted in lethal immunopathology [26]. In in vitro experiments, type I IFNs skewed *Candida*-induced inflammation from a Th17-response toward a Th1-response suggesting that the type I IFN pathway is a main signature of *Candida*-induced inflammation [30]. Type I IFN signaling was also reported to be detrimental for host defense against *C*. *glabrata* [27] and *Histoplasma capsulatum* [24] but protective in mice infected with *Aspergillus fumigatus* [25,31]. Therefore, the innate antiviral inflammatory response against HIV infection associated with type I IFNs could also dramatically impact on the ability of the host to contain opportunistic infections, but this possibility has received much less attention.

Polyinosinic-polycytidylic acid (poly-IC), a double-stranded RNA (dsRNA) virus mimic, can be used in experimental models to stimulate enhanced production of type I IFNs. Poly-IC condensed with poly-L-lysine and carboxymethylcellulose (pICLC, Hiltonol) is a stabilized version of poly-IC, which is designed to stimulate prolonged, high-level production of type I IFN [32,33] and is available for investigation in humans [34]. Ds RNA can be recognized by two major pattern recognition receptors, Toll-like receptor 3 (TLR3) and the melanoma differentiation-associated protein-5 (MDA5) [34]. TLR3 is located in the endosomal compartment and senses the dsRNA following its internalization through endocytosis, whereas MDA5, which is a cytosolic sensor protein, recognizes the pICLC that penetrates into the cytosol [35,36]. Upon pICLC recognition, TLR3 signaling occurs by recruiting the adaptor protein TRIF (Toll/IL1 resistance domain-containing adaptor inducing IFNβ), whereas MDA5 associates with the adaptor protein IPS1 (IFNβ promoter stimulator 1) [37,38]. Both of these adaptors initiate downstream signaling via activation of the transcription factors, IRF3 and IRF7, which in turn induce the expression of genes encoding type I IFN and various proinflammatory cytokines [39].

In this study, we investigated the effect of high type I IFN environments on the cellular innate and adaptive immune responses to *C*. *neoformans* and the outcome of infection. We found that type I IFN induced by pICLC was highly protective in C57BL/6 mice infected with *C*. *neoformans* via MDA5 and not TLR3 recognition. In addition, we demonstrated that pICLC combined with fluconazole (FLC) resulted in a synergistic anti-fungal effect reflected in reduction of fungal loads. At the cellular level, pICLC administration induced a massive influx of Ly6C^high^ monocytes and neutrophils into the lung tissue parenchyma within 3 days and sustained their elevated numbers for weeks. Moreover, pICLC treated mice displayed diminished GATA3+ IL-5 producing CD4 T cells and dramatically reduced eosinophilia, but increased RORγt^+^ IL-17A producing CD4 T cells. Importantly, pICLC induced protection against *C*. *neoformans* required CD4 T cells, IFNγ, and to a lesser extent IL-17A. Lastly, we found that pICLC treatment also enhanced host resistance against *C*. *gattii* infection. These data show that the induction of high levels of type I IFN can reorganize the innate and adaptive immune responses against cryptococcal infection, illustrating how manipulation of specific inflammatory responses can dramatically improve resistance to infection with etiologic agents of cryptococcosis.

## Results

### PICLC protects against *C*. *neoformans* infection via the MDA5-IFNα-IFNaR axis

**Fig 1:**
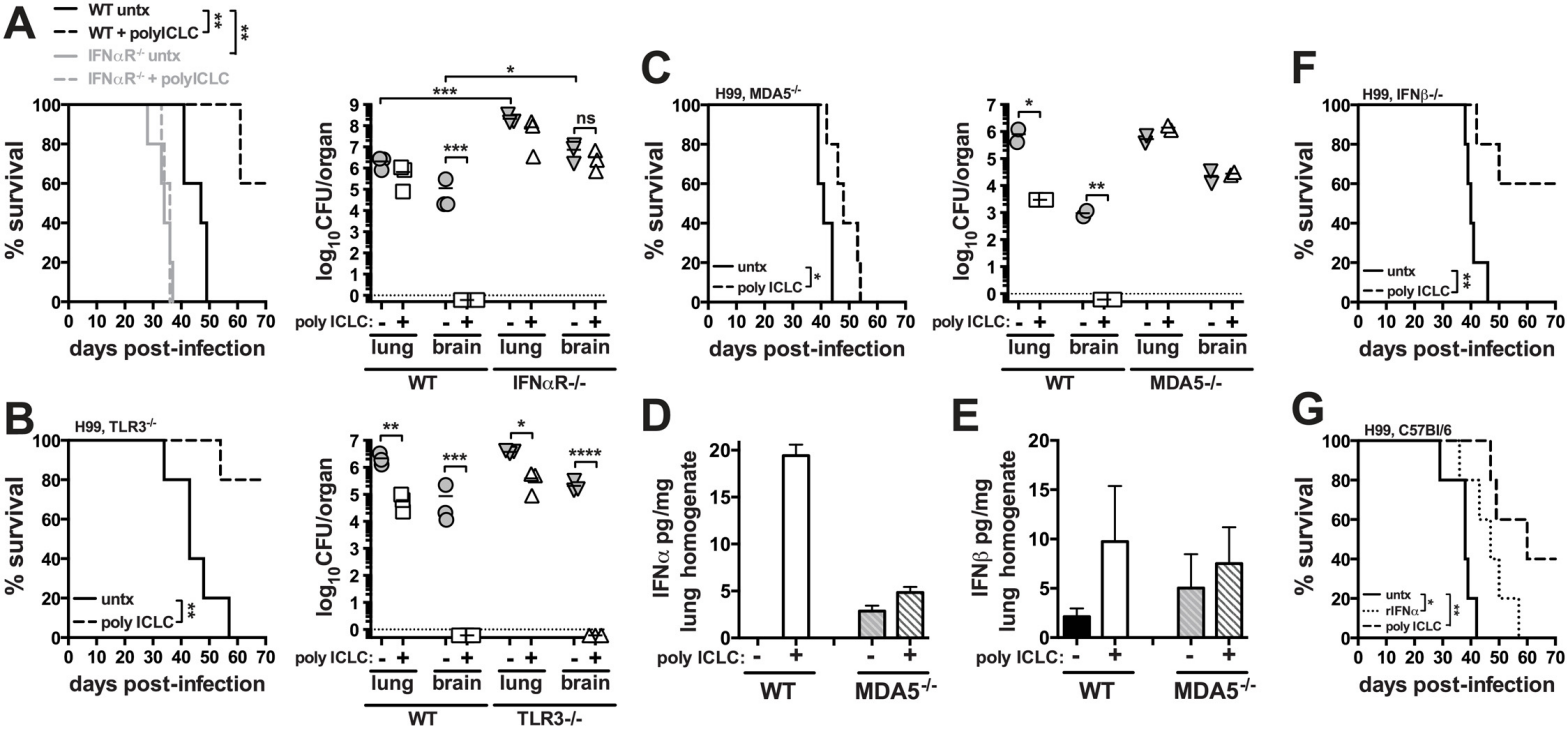
Treatment of mice with pICLC protects against *C*. *neoformans* infection through the MDA5-dependent induction of type I interferon. Mice were intrapharyngeally infected with 5000 CFU of *C*. *neoformans* H99 and treated with polyICLC twice weekly starting on the day of infection or left untreated. Survival and fungal burdens on day 28 post-infection in WT and *Ifnar1* ^*-/-*^ (**A**), *Tlr3* ^*-/-*^ (**B**), and *Mda5* ^*-/-*^ mice (**C**). IFNα (**D**) and IFNβ (**E**) concentrations in lung homogenate were measured by ELISA on day 28 post-infection. Cytokine concentrations were normalized to the total content of protein in the sample. (**F**) Survival of treated or untreated *Ifnb* ^*-/-*^ infected mice. (**G**) WT mice were infected and either given no treatment or treated with pICLC or rIFNα and monitored for survival. Data are representative of at least 2 independent experiments with n = 5–6 mice/group. *P* values for comparisons of fungal burdens are calculated based on log_10_ transformed values.

To investigate the impact of innate inflammation associated with viral infections on the outcome of cryptotococosis, C57BL/6 or mice deficient in type I interferon receptor signaling (*Ifnar1* ^*-/-*^) were intrapharyngeally exposed to *C*. *neoformans* and treated twice weekly with pICLC, a stabilized dsRNA virus mimic. While all of untreated WT mice succumbed to infection by day 49, 60% of treated WT mice were remaining at the end of the experiment on day 70 post-infection (Fig 1A). On day 28 post-infection, the pulmonary fungal burden of pICLC treated WT mice was reduced in the lungs by ~0.5 logs (Fig 1A). Strikingly, while the control mice had ~10^5^ CFU in the brain, the CFU in the brains of treated mice was barely detectable (Fig 1A). Untreated *Ifnar1* ^*-/-*^ mice succumbed to infection significantly earlier and had a nearly 2 log increase in fungal loads in the lungs and brain compared to untreated WT mice while pICLC administration had no effect on the survival or fungal control of H99-infected *Ifnar1* ^*-/-*^ mice (Fig 1A). These results indicate that type I interferon plays a major role in normal host resistance to H99 infection and that inducing high levels of type I interferon via pICLC administration further enhances its potent protective effects. Histopathological analysis showed that ~70% of lung parenchyma of untreated mice was affected compared to 25–30% of lung parenchyma in pICLC-treated animals (S1 Fig). PICLC did not affect fungal growth in YPD broth indicating that the antifungal effects in vivo were not due to direct activity against the yeast. Collectively, these results suggest that inflammation associated with innate recognition of dsRNA, which simulates a viral infection, leads to a profound resistance to invasive pulmonary cryptococcal infection.

Pattern recognition receptors TLR3 and MDA5 have been implicated in the recognition of dsRNA, so we next treated *C*. *neoformans* H99 infected *Tlr3* ^*-/-*^ and *Mda5* ^*-/-*^ mice with pICLC and monitored survival and fungal growth. Administration of pICLC significantly extended survival of *C*. *neoformans* infected *Tlr3* ^*-/-*^ mice (Fig 1B; *P*<0.05), and similar to the WT mice, fungal growth was nearly undetectable in the brain of pICLC treated TLR3 KO mice (Fig 1B). In contrast, pICLC treatment of H99 infected *Mda5* ^*-/-*^ mice had insignificant effects on survival and no detectable effect on fungal burden (Fig 1C). Furthermore, we observed a significant induction of IFNα (Fig 1D; *P*<0.01) and moderate induction of IFNβ (Fig 1E) in lung tissue homogenates of pICLC treated WT but not *Mda5* ^*-/-*^ mice. Collectively, these data indicate that MDA5 recognition of pICLC is required for the increased induction of type I IFN and the protective effects of pICLC against cryptoccocal infection.

To differentiate between the roles of IFNα and IFNβ we investigated the effect of pICLC administration on survival of *Ifnb* ^*-/-*^ mice following infection with *C*. *neoformans*. Importantly, pICLC treatment protected IFNβ deficient mice infected with H99 (Fig 1F), suggesting a possible role of IFNα in the pICLC induced protection against cryptococcal infection. Administration of recombinant mouse IFNα to H99-infected WT mice slightly extended survival (Fig 1G) while rIFNβ treatment did not delay mortality of the mice compared to the untreated control (S2 Fig). Together, these data indicate that MDA5 mediated recognition of pICLC induces IFNα which signals through IFNaR1 to induce control of *C*. *neoformans* infection.

**Fig 2:**
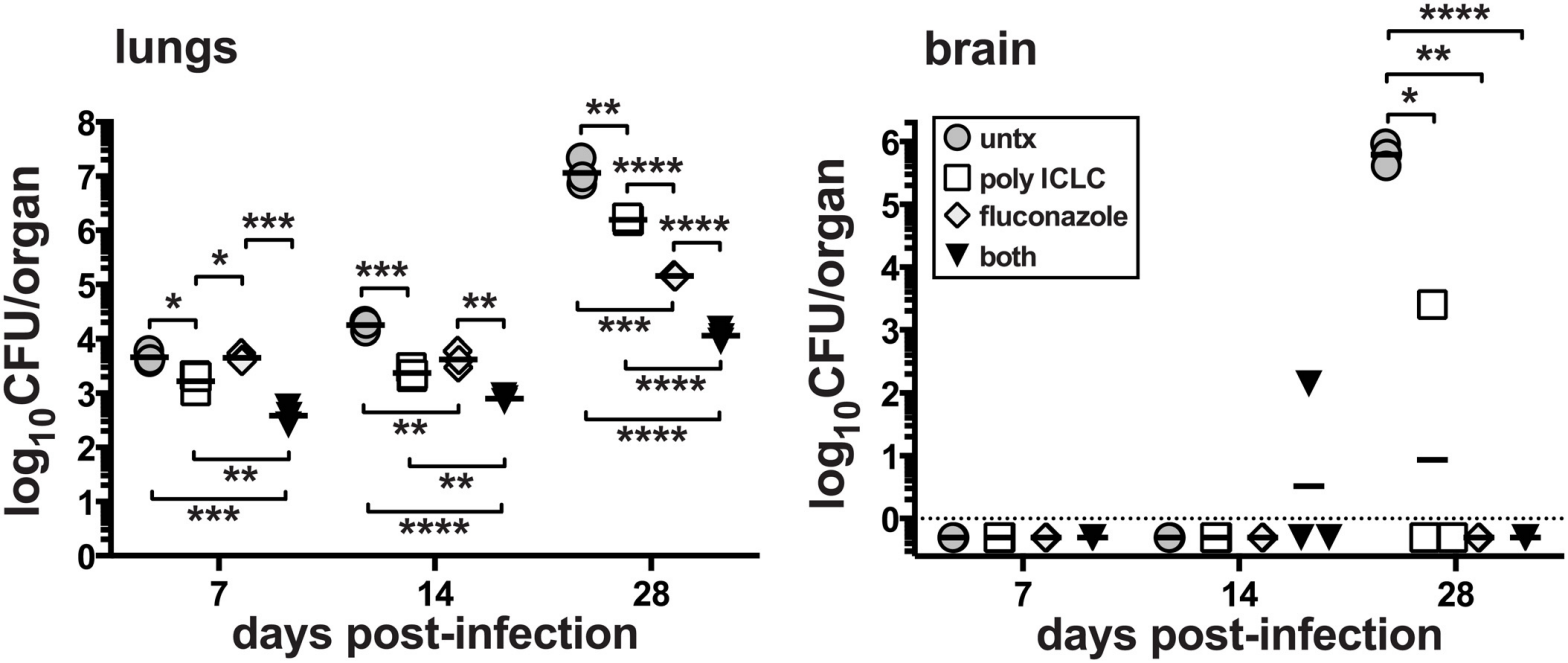
Treatment of mice with pICLC synergizes with FLC to protect against *C*. *neoformans*. Mice were intrapharyngeally infected with 5000 CFU of *C*. *neoformans* H99 and left untreated, intrapharyngeally administered pICLC, twice weekly, treated with fluconazole via daily intraperitoneal injections, or given both treatments concomitantly. Treatments were started 24 h after infection. Brains and lungs were harvested on days 7, 14, and 28 to measure fungal burdens. Data are representative of 3 experiments with n = 3–5 mice/group.

We next asked if pICLC could enhance standard antifungal therapy of *C*. *neoformans* infection. Mice were intrapharyngeally infected with H99 and treated with pICLC, FLC, or both, and CFU were quantified in the lungs and brain on days 7, 14 and 28. While monotherapy with either FLC or pICLC had a significant effect on fungal growth, co-treatment with the antifungal drug and immunomodulatory therapy together further decreased fungal replication (Fig 2). Thus, the protective effects of IFNaR1 driven innate inflammation can further enhance standard antifungal therapy.

### PICLC treatment drives rapid influx of neutrophils and Ly6C^high^ monocytes into the lung tissue parenchyma and suppresses eosinophilia

**Fig 3:**
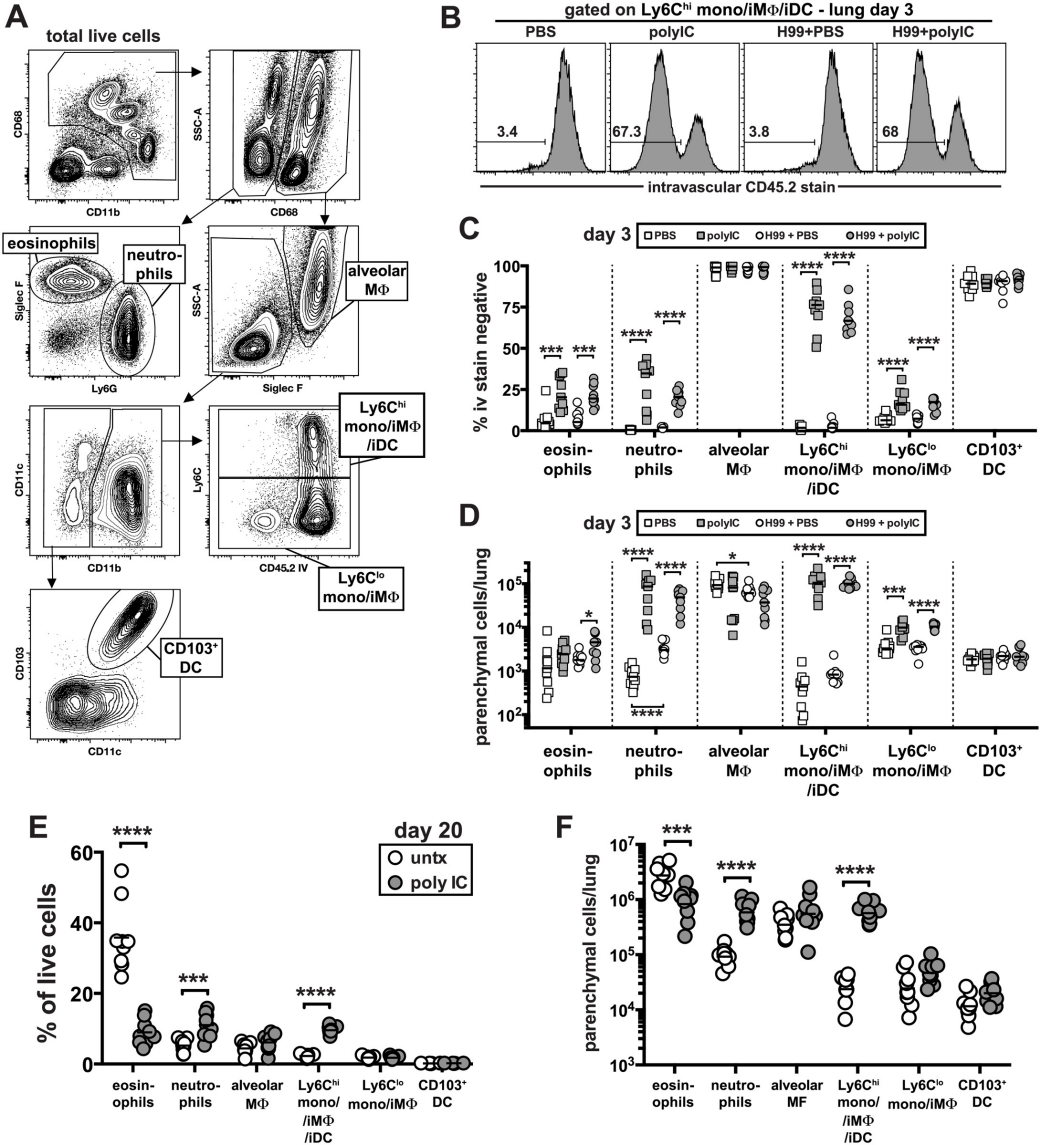
Delivery of pICLC into the airways induces the rapid influx of neutrophils and Ly6C^high^ monocytes into the lung tissue parenchyma and suppresses *C*. *neoformans* induced eosinophilia. (**A**) Gating strategy used in these experiments to identify distinct myeloid cell subsets. Example plots are taken from the lungs of a naïve animal. (**B-D**) Naïve and H99 infected mice were intrapharyngeally administered PBS or pICLC once at time of infection and myeloid cell responses were analyzed on day 3. To discriminate intravascular and parenchymal leukocytes, mice were intravenously injected with anti-CD45.2-FITC and euthanized 3 minutes later. (**B**) Example FACS plots of the intravascular stain after gating on Ly6C^high^ monocytes as shown in (A). Percentage (**C**) and absolute number (**D**) of each myeloid cell type that is intravascular stain negative in the lung. Data in (**B-D**) on day 3 post-infection are pooled from 2 separate experiments with n = 5 mice/group. (**E**) The percentage of total live lung cells that are each myeloid cell type in untreated and pICLC treated mice on day 20 post-infection. (**F**) Number of each myeloid cell type in the lungs that is intravascular stain negative on day 20 post-infection. Data were pooled from two independent experiments with n = 4–5 mice/group.

We next examined the early and later changes in the cellular innate immune response in the lung following infection with and without pICLC treatment. To do so, we identified six major populations of pulmonary myeloid cells by multi-color flow cytometry: alveolar macrophages, neutrophils, eosinophils, Ly6C^high^ monocyte/macrophages, Ly6C^low^ monocyte/macrophages, and CD103^+^ dendritic cells (Fig 3A). To carefully quantify the migration of myeloid cell types into the lung tissue parenchyma, we employed the well-characterized intravascular staining approach that allows for the flow cytometric discrimination of parenchymal and intravascular cells [40]. Three minutes before euthanasia and lung harvest, the mice were intravenously (iv) injected with a fluorochrome labeled-mAb against CD45. Cells staining positive for the iv injected antibody were located in the lung blood-associated vasculature, and the cells that were protected from the iv stain were localized to the lung parenchyma (Fig 3B). In all groups of mice the alveolar macrophages and CD103^+^ dendritic cells were almost exclusively localized to the lung parenchyma as expected for these tissue resident cell types (Fig 3C). The percentage of eosinophils that were iv stain negative also increased but due to their low abundance at this early time point this only resulted in a ~2 fold increase in the number of eosinophils in the lung parenchyma of infected mice following pICLC treatment (Fig 3D). In contrast, the frequency of iv stain negative neutrophils increased dramatically in uninfected as well as pICLC treated mice, resulting in an ~120 fold increase in the number of neutrophils in the lung parenchyma (Fig 3C and 3D).

Consistent with the increased migration of neutrophils, pICLC treatment induced a large increase in KC and G-CSF expression compared to untreated controls, which was completely IFNaR1 dependent (S3A and S3B Fig). Interestingly, the only cell type to appreciably change following infection alone at day 3 were the neutrophils, which increased ~8 fold in the lung tissue parenchyma (Fig 3D).

PICLC induced the most dramatic change in Ly6C^high^ monocytes/macrophages. In naïve mice, <5% of these cells were iv stain negative, and infection with H99 did not significantly increase their localization into the lung parenchyma (Fig 3C and 3D). Following administration of pICLC to naïve or H99 infected mice, ~70% of the Ly6C^high^ monocytes/macrophages were now iv stain negative corresponding to an ~240 fold increase in the number of these cells in the lung tissue parenchyma (Fig 3B–3D). Ly6C^high^ monocytes are well understood to express high levels of CCR2, and accordingly we observed a large induction of the ligand CCL2 in pICLC treated mice, which was also IFNaR1 dependent (S3C Fig).

In contrast to day 3 when there were few eosinophils and the majority were in the lung associated blood-vasculature, by day 20 post-infection ~30% of all live cells were eosinophils (Fig 3E) and >90% were within the lung parenchyma (S4 Fig). In contrast, in pICLC treated mice fewer than 10% of the cells isolated from the lung were eosinophils. The percentage of eosinophils that were localized to lung tissue parenchyma, however, was not decreased (S4 Fig), but the total number of eosinophils in the lung parenchyma was reduced by ~3 fold (Fig 3F).

These data suggest that pICLC treatment may have inhibited the production of eosinophils but not their ability to migrate into the lung tissue. Similar to what was observed at day 3, on day 20 post-infection we found a large increase in the abundance of neutrophils and Ly6C^high^ monocytes/macrophages along with a dramatic increase in the number of lung parenchymal cells (Fig 3E and 3F). Collectively, these data show that intrapharyngeal administration of pICLC induces an extremely rapid and sustained influx of neutrophils and Ly6C^high^ monocytes/macrophages into the lung parenchyma, and suppresses the development of eosinophilia.

### PICLC treatment of *C*. *neoformans* infected mice skews CD4 T cell polyfunctionality toward Th17 responses

**Fig 4:**
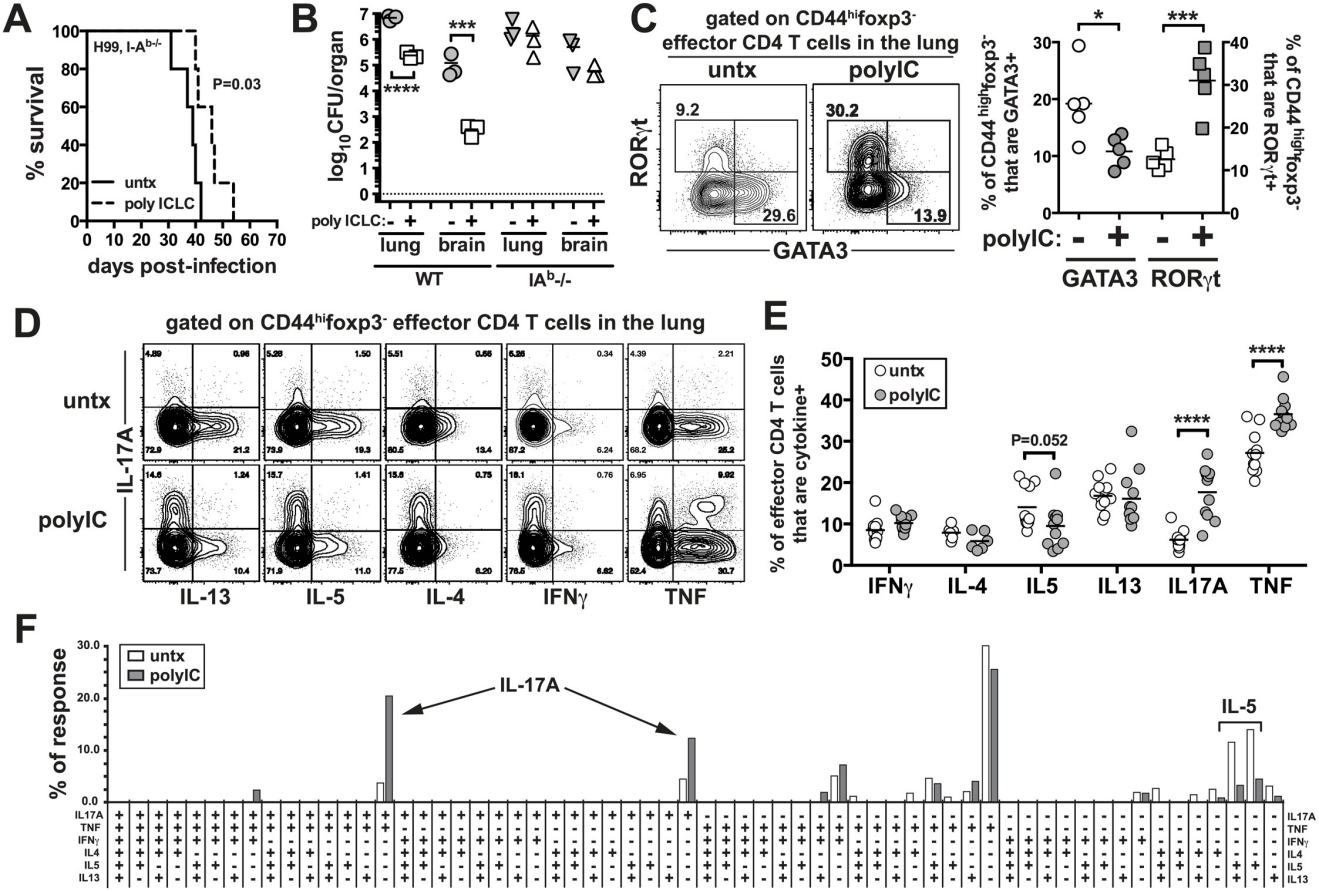
PICLC treatments enhances Th17 responses in *C*. *neoformans* infected mice. *I-A* ^*b-/-*^ mice were intrapharyngeally infected with 5000 CFU of H99 and either left untreated or given pICLC and monitored for survival **(A)** or sacrificed on day 27 to measure fungal burdens in the lungs and brains **(B)**. Data are representative of three independent experiments with n = 5 mice/group. **(C)** RORγt and GATA-3 intracellular staining in CD44^high^foxp3^-^ effector CD4 T cells isolated from the lungs of treated and untreated mice on day 20 post-infection. Example FACS plots **(D)** and summary data **(E)** of intracellular cytokine production by lung CD44^high^foxp3^-^ effector CD4 T cells following restimulation with anti-CD3/anti-CD28 on day 28 post-infection. Data are pooled from 2 separate experiments with n = 5 mice/group. **(F)** Cytokine polyfunctionality of effector CD4 T cells in the lungs of untreated or pICLC treated mice on day 28 post-infection. Data are pooled from 2 separate experiments with n = 5 mice/group. *P* values for comparisons of fungal burdens are calculated based on log_10_ transformed values.

We next asked what role CD4 T cells play in the pICLC induced protection, by treating H99 infected *I-A* ^*b-/-*^ mice with pICLC. While there was a tendency for delayed mortality in pICLC treated CD4 T cell deficient mice compared to untreated mice, this difference was not statistically significant and pICLC had no effect on fungal loads in the absence of CD4 T cells (Fig 4A and 4B), indicating that CD4 T cells are required for protection in pICLC treated H99 infected mice. Therefore, we next analyzed the effects of pICLC treatment on the differentiation of CD4 T cells in infected mice. We found that pICLC suppressed the expression of the GATA-3 and enhanced the expression of RORγt in foxp3^-^CD44^hi^ effector CD4 T cells (Fig 4C), indicating that pICLC treatment skews the CD4 T cell response against H99 from a Th2 towards a Th17 response. To characterize the function of pulmonary CD4 T cells, we simultaneously measured the production of IL-17A, IL-4, IL-5, IL-13, IFNγ, and TNF by polychromatic intracellular cytokine staining following re-stimulation with anti-CD3/28 in vitro. We found that the overall frequency of CD4 T cells capable of producing IL-17A was ~3 fold higher in the pICLC treated compared to untreated mice (Fig 4D and 4E). Moreover, there was a trend towards a reduction in the frequency of IL-5 producing CD4 T cells just below statistical significance (Fig 4D and 4E).

In order to determine if the increase in IL-17A^+^ CD4 T cells was due to an increase in Th17 cells themselves or its production by Th1 or Th2 cells, we quantified the frequency of CD4 T cells producing all possible combinations of these six cytokines. We found that the increase in IL-17A^+^ CD4 T cells in pICLC treated mice was due to increases in cells producing IL-17A alone or cells making only IL-17A and TNF (Fig 4F).

Therefore, Th1 and Th2 cells did not begin to make IL-17A in pICLC treated mice, and the increase in IL-17A producing CD4 T cells in pICLC treated mice was likely due to the expansion of bona fide Th17 cells. We also measured the parenchymal localization of effector CD4 T cells using the intravascular stain. We found that pICLC increased the frequency of CD44^high^foxp3^-^ effector/memory phenotype CD4 T cells that were within the parenchyma from ~85% in the untreated to ~95% in the treated (S5 Fig). Collectively, these results show that pICLC treatment of H99 infected mice skews the CD4 T cell response away from detrimental Th2 cells and towards protective Th17 cells enhancing the migration of effector T cells into lung tissue.

### IFNγ and IL-17A contribute to the anti-cryptococcal protective effects of pICLC treatment

**Fig 5:**
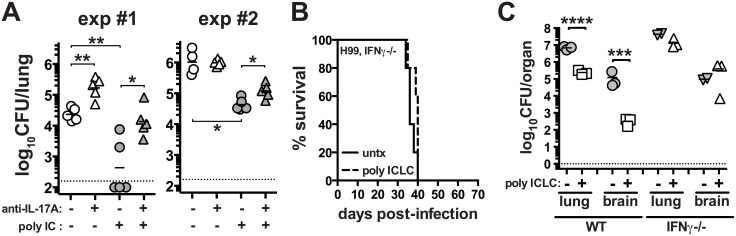
IL-17A and IFNγ contribute to pICLC induced protection against *C*. *neoformans* infection. (**A**) WT mice were intrapharyngeally infected with 5000 CFU of H99 and either left untreated, given pICLC, anti-IL-17A, or both pICLC and anti-IL-17A concomitantly. Lungs were harvested on day 20 and fungal burdens were quantified. Results from 2 independent experiments are shown separately. *Ifng* ^*-/-*^ mice were intrapharyngeally infected with 5000 CFU of H99 and either left untreated or given pICLC and monitored for survival (**B**) or sacrificed on d28 to quantify fungal burdens in the lungs and brains **(C)**. Data are representative of 3 independent experiments with n = 3–5 mice/group. *P* values for comparisons of fungal burdens are calculated based on log_10_ transformed values.

Given the enhanced production of Th17 cells following pICLC administration to H99 infected mice, we asked if IL-17A itself was contributing to the protective effects of the treatment. H99 infected mice were treated with and without pICLC and with and without anti-IL-17A neutralizing mAb. We found that in two independent experiments IL-17A blockade increased the fungal loads in the pICLC treated mice (Fig 5A). These data indicate that IL-17A is partially involved in the control of H99 infection in pICLC treated mice. Although there was no major increase in IFNγ-producing CD4 T cells after pICLC administration (Fig 4E), we observed increased levels of IFNγ in BAL fluid on day 7 upon pICLC administration to H99 infected mice (S3D Fig) indicating that the increased levels of IFNγ in treated mice may have originated from other cellular sources. We also found that IFNγ-producing CD8 T cells were not significantly increased, indicating that an innate lymphocyte such as NK cells may be the cell type responsible for the elevated levels of IFNγ in the polyICLC treated mice. Since IFNγ is known to play a key role in host defenses against *C*. *neoformans* [41], we examined the requirements for this cytokine in the protective effects of pICLC by treating H99 infected *Ifn*g^-/-^ mice. Indeed, we found that pICLC treatment did not rescue IFNγ deficient mice as the animals succumbed to infection at the same rate as untreated controls (Fig 5B). In addition, there was little or no suppression of fungal growth in pICLC treated IFNγ deficient mice (Fig 5C). Taken together, these data suggest that both IL-17 and IFNγ are required for pICLC mediated protection against *C*. *neoformans*.

### PICLC protects mice from pulmonary infection with *Cryptococcus gattii*

**Fig 6:**
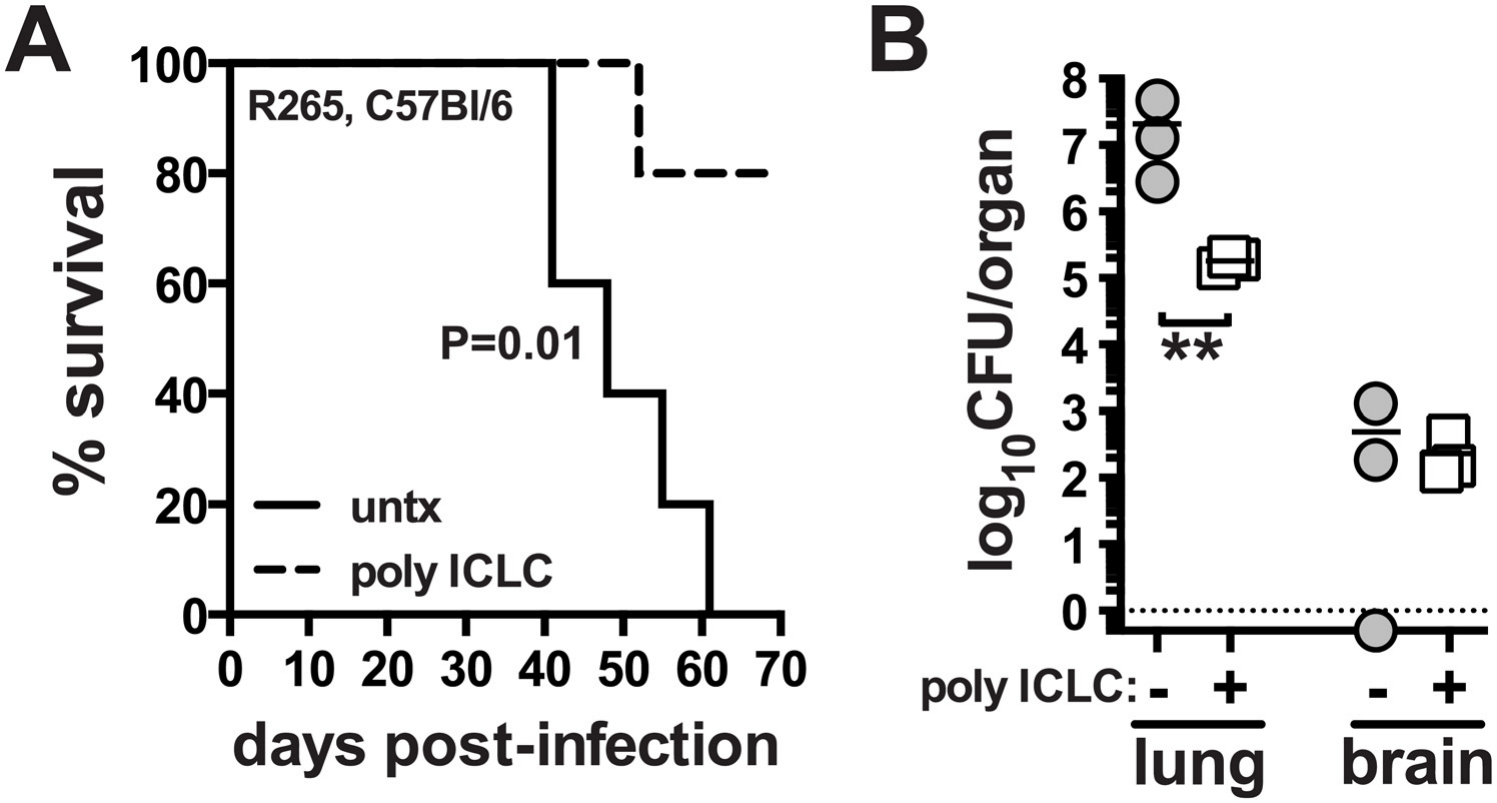
PICLC treatment protects against pulmonary infection with *C*. *gattii*. WT mice were intrapharyngeally infected with 5000 CFU of *C*. *gattii* R265 and either left untreated or given pICLC and monitored for survival **(A)** or sacrificed on day 28 to measure fungal burdens in the lungs and brains **(B)**. Data are representative of 3 experiments with n = 5–6 mice per group. *P* values for comparisons of fungal burdens are calculated based on log_10_ transformed values.

We next asked if pICLC treatment could also enhance host resistance to *C*. *gattii*, another etiologic agent of cryptococcosis. We chose strain R265, which primarily induces lethal pneumonia in mice, as opposed to *C*. *neoformans* which causes lethal meningoencephalitis by hematogenous dissemination from the lungs and fulminating growth in the central nervous system [42]. Mice were infected with R265 and subjected to the same pICLC treatment regimen. We found that pICLC significantly increased host survival after infection, with all untreated mice succumbing by ~day 60 while 80% of the treated mice still remaining on day 70 (Fig 6A). *C*. *gattii* grows much less in the brain compared to H99, and pICLC treatment had little effect on fungal loads in the brain. However, there was close to a 2 log reduction in the fungal loads in the lungs of treated compared to untreated mice (Fig 6B). These data show that the host-protective effects of pICLC triggered inflammation extend to both *C*. *neoformans* and *C*. *gattii* despite their difference in organ tropism and end-stage disease manifestation.

## Discussion

Cryptococcocal co-infections are among the most important AIDS-associated opportunistic infections. However, the underlying immunological mechanisms that predispose HIV+ patients to cryptococcosis might not solely be attributable to CD4 depletion. In the present study we investigated the effect of type I IFN induction, a type of innate inflammation typically associated with viral infections, on the outcome of cryptococcosis by employing a dsRNA homolog, pICLC, as the type I IFN inducing agent to mimic viral co-infection.

PICLC treatment induced multiple changes in both innate and adaptive cellular immune responses in H99 infected mice. This is not unexpected since poly-IC challenge is known to elicit gene expression changes in innate and cell mediated immune pathways similar to acute viral infection [43,44]. In the first three days after infection, we observed very little recruitment of myeloid cells into the lungs with this strain, regardless of dose and route of *C*. *neoformans* inoculation. The only noticeable change at the early time point after infection was a minor increase in the number of neutrophils in the lungs. This suggested that the yeast cells were able to persist and replicate in the lungs for at least 3 days before appreciable numbers of circulating phagocytes are recruited to the site of infection. Of note, a single administration of pICLC into the airways induced an enormous recruitment of myeloid cells in this time period, even without any infection. We found a >100 fold increase in the number of neutrophils and Ly6C^high^ monocytes in the lungs three days after pICLC treatment.

Early studies have reported conflicting results regarding the effect of poly-IC in mice infected by *C*. *neoformans*. While it was protective in one study [45], it had no effect on infection in the other [46]. The reason for these different results is unclear since both studies used a similar protocol and for obvious reasons, no immunological mechanisms were addressed. In addition, mice in those studies had been infected intravenously with at least a1000-fold higher inoculum of an uncharacterized or a serotype A *C*. *neoformans* strain and used poly-IC instead of pICLC. We did not observe a beneficial effect of pICLC in mice infected intravenously with H99 (S6 Fig). Importantly, the route of infection and the size of inoculum, as well as the *C*. *neoformans* strains used are all critical for the outcome of experimental cryptococcosis in an animal model [42]. One could speculate that during infection, the events mediated by pICLC may help the host to keep cryptococci contained in the lung but once the fungus is blood borne these events are unable to confer protection. This notion is supported by the fact that pICLC protected mice from meningoencephalitis only if it was administered within 72 hours post intrapharyngeal inoculation of *C*. *neoformans*. Once the cryptococci inoculated intrapharyngeally enter the brain, which occurs by 72h [42], pICLC fails to control cryptococcal growth.

Poly-IC is recognized by both TLR3 [34,47] and MDA5 [34,48] but our results in this study indicate that pICLC administered to mice infected with cryptococci was recognized by MDA5 and not by TLR3. However, our data do not rule out the possibility that specifically triggering TLR3 by other means might also be protective against cryptococcal infection. Interestingly, Jaeger and coworkers recently reported that MDA5 is directly involved in the host defense against *Candida* infection but the active ligands for MDA5 in this context are not clear [49]. Poly-IC has also been tested in mice infected with *Candida albicans* [50,51] and *Aspergillus fumigatus* [25] and shown to have either a detrimental or a beneficial effect in mice, respectively. Results obtained with poly-IC corroborated the differential effects of type I IFN signaling observed in *Candida* [26] and *Aspergillus* infected hosts [25], respectively. Guarda and colleagues demonstrated that the poly-IC induced type I IFN increased susceptibility to *C*. *albicans* infection by diminishing IL-1β production [51]. In the case of pulmonary *H*. *capsulatum* infection, pre-treatment with poly-IC had no effect on early fungal growth [52]. The poly-IC recognition receptor has not been identified in these studies [25,50]. In fact, our study is the first to identify the pattern recognition receptor for a dsRNA virus mimic in a host co-infected with pathogenic fungi.

It is important to point out that we used the intravascular staining approach to formally show that these myeloid cells had migrated out of circulation and were located in the lung tissue parenchyma, and the enhanced numbers of parenchymal neutrophils as well as monocytes in pICLC treated mice were sustained for at least 3 weeks post-infection. It has been shown that recruitment of Ly6C^high^ monocytes into the lungs following exposure to *C*. *neoformans* is associated with control of the infection and is dependent on CCR2 [53,54] and mice deficient in CCR2 are much more susceptible to *C*. *neoformans* infection [55]. Future studies of individual contributions of each of these cellular changes in the anti-cryptococcal effect of pICLC may provide further insight into the qualities of a protective innate immune response against cryptococcosis.

PICLC treatment of H99 infected mice also greatly altered the polyfunctional profile of the pulmonary CD4 T cell response. CD4 T cells in the lungs of these mice showed reduced IL-5 production and GATA-3 expression. It has been shown that IL-5 produced by CD4 T cells is required for eosinophilia in the context of cryptococcal infection [56] and IL-5 overexpressing mice are highly susceptible to *C*. *neoformans* infection [57]. Indeed, we found that pICLC treatment greatly suppressed the development of eosinophilia normally observed in *C*. *neoformans* infection, indicating that the effect on eosinophil numbers in the lungs may be due to the impact of pICLC on CD4 T cell effector polarization in *C*. *neoformans* infected mice. Interestingly, pICLC administration also greatly increased Th17 cell generation in H99 infected mice. Consistent with recent reports in mice infected with *C*. *neoformans* H99 [58] or the less virulent strains 52D [59] or H99γ [60] where some protective role for IL-17A was observed [59], we found that IL-17A induction by pICLC was partly responsible for control of the infection. In addition to the known function of IL-17 in neutrophil recruitment [61], Th17 stimulation may also enhance the ability of alveolar macrophages to phagocytize and control the intracellular proliferation of cryptococci, as reported using in vitro studies with human primary macrophages [62]. The mechanisms of pICLC induction of RORγt Th17 cells are not clear, but the increase in Th17 responses in treated mice may result from the decreased levels of counter-regulatory GATA-3. More detailed studies are needed to specifically address the mechanisms of Th17 induction in this setting.

The clinical evidence in AIDS associated cryptococcosis unequivocally demonstrates that CD4 T cell mediated immunity is paramount to the host resistance to cryptococcosis [8]. The susceptibility of HIV+ individuals to opportunistic *C*. *neoformans* infection, therefore, is thought to be primarily due to CD4 T cell depletion [4,8]. Recent studies, however, showed that high type I IFN environments can exacerbate bacterial and other fungal infections often associated with HIV+, such as *M*. *tuberculosis* [21,63,64] histoplasmosis [24] and candidiasis [26]. These reports have led to the idea that the innate inflammation induced by the chronic viral infection might also be a major factor predisposing to both primary and opportunistic infections in which high level induction of type I IFN may be detrimental for infection outcome.

Our results clearly indicate that type I IFN signaling plays an important role in defending the host from cryptococci and helps integrate early, innate immune responses with later events mediated by the adaptive immune system. We found that *Ifnar1* ^*-/-*^ mice are highly susceptible to H99, and furthermore, triggering type I IFN through the administration of pICLC induced multiple immunological changes leading to a profound resistance to this fungal infection. In agreement with our findings, one previous study found that IFNα receptor knockout mice displayed defective Th-1 responses following exposure to *C*. *neoformans* [65]. These results initially seemed inconsistent with the well-established susceptibility of HIV infected individuals to *C*. *neoformans*. However, we also found that pLCLC-mediated protection was dependent on CD4 T cells. It is therefore possible that the type I IFN response induced by HIV infection is protective against *C*. *neoformans* only until CD4 T cells fall below a critical threshold after which the protective effects are lost and the lack of CD4 T cells results in uncontrolled infection. Indeed, cryptococcosis is usually observed in late stage HIV infection when CD4 T cell numbers are extremely low [8].

The critical role for CD4 T cells in the pICLC mediated protection may suggest limited therapeutic implications of type I IFN based therapies for cryptococcosis in AIDS patients, as the most of the HIV-associated cryptococcosis occur as the patients become deficient for CD4 T cells [8]. On the other hand, type I IFN based therapies may be a possibility for cryptococcosis caused by *C*. *gattii* since we found pICLC treatment to be very effective against *C*. *gattii* infection which occurs more frequently in immunocompetent individuals rather than CD4 T cell depleted immunocompromised patients [66]. PICLC treatment had synergistic effect with FLC, the most widely used azole for the treatment of cryptococcosis [67]. When administration of the dsRNA was initiated 24 hours post infection, the therapeutic efficacy of pICLC alone was higher than FLC alone; however, combination of the two was significantly more effective than either one of them alone. These results implicate a potentially prophylactic importance of FLC combined with pICLC in HIV+ patients prior to decrease in CD4 T cell count. Regardless of the prophylactic/therapeutic potential of type I IFN specifically, these data serve as a proof of concept that manipulation of innate inflammatory signals can dramatically improve host resistance to cryptococcal infections caused by both agents of cryptococcosis, *C*. *neoformans* and *C*. *gattii*.

## Materials and Methods

### Mice

WT C57BL/6 mice were purchased from DCT (National Cancer Institute, NCI-Frederick, MD). *Ifnabr* (*Ifnar1* ^*-/-*^ *)*, *Tlr3* ^*-/-*^, *Ifng* ^*-/-*^ and *I-A* ^*b-/-*^ (Taconic:C57BL/6NTac-[KO]AbB) on a C57BL/6 background, were purchased from Taconic Farms (Germantown, NY) under the NIAID Animal Supply Contract. *Mda5* ^*-/-*^ mice were obtained from The Jackson Laboratory. *Ifnb* ^*-/-*^ mice [68] were kindly provided by Dr. Stephanie N. Vogel (University of Maryland, Baltimore). Male and female mice between 8 and 12 weeks of age were used in all experiments.

### Fungal strains and media

The strains H99 and R265, both genome sequenced strains were chosen as representative strains of *C*. *neoformans* and *C*. *gattii*, respectively. Strains were stored in 25% glycerol at −80°C until use and were maintained on YEPD (1% yeast extract, 2% peptone, 2% glucose) agar plates at 30°C.

### Murine infection model

To induce respiratory infection, WT and knockout mice were anesthetized with isoflurane and inoculated with the designated number of yeast cells in 20 μl of phosphate-buffered saline (PBS) via intrapharyngeal (i.p.) aspiration (inhalation method) [69,70]. Each mouse received approximately 5000 colony forming units (CFUs) of *C*. *neoformans* or *C*. *gattii*. Survival was monitored for up to 80 days post inoculation. Mice were observed daily for signs of disease and lethality. Mice with signs of irreversible disease (e.g., persistent hunching, unsteady gait, lethargy, unable to eat or drink) were euthanized. For quantification of fungal burden, organs (lung and brain) were weighted, homogenized, and diluted in 10-fold steps in PBS. Fungal CFUs were determined by plating serially diluted homogenates on YEPD agar plates. Lungs and brains from mice were subjected to histopathologic staining with hematoxylin and eosin (H&E) and Gomori methenamine silver (GMS).

### Poly-ICLC and in vivo treatment

PICLC (Hiltonol), a synthetic analogue of viral dsRNA, was supplied by Oncovir Inc. Mice were inoculated intrapharyngeally with pICLC (5 μg in 20 μl/mouse) twice weekly up to 10 weeks, starting at the same day of infection. To examine preventive or therapeutic effect pICLC was administered twice weekly up to 10 weeks, starting 3 days prior or 1 day after cryptococcal exposure, respectively. Recombinant mouse IFNα and IFNβ were generous gifts from PBL InterferonSource (Piscataway, NJ). IFNα or IFNβ (1000 U) was administered intrapharyngeally twice weekly up to 10 weeks, starting at the same day of infection. Fluconazole (FLC) was used along with pICLC to assess antifungal combination therapy. FLC was administered intraperitoneally at a concentration of 10 mg/kg of body weight/day, starting 24 h after infection and continued for 28 days. For IL-17A neutralization experiments, mice were injected with 200 μg of αIL-17A mAb clone 17F3 every three days.

### Intravascular stain and leukocyte isolation

The intravascular staining was performed as described previously [71]. Briefly, mice were injected i.v. with 2.5 μg of αCD45.2-FITC mAb in a volume of 200 μl, and after 3 minutes the mice were euthanized and the lungs were harvested. To isolate pulmonary leukocytes lungs were minced with scissors and shaken in a cocktail of DNase, collagenase and hyaluronidase for ~30 minutes at 37°C. The leukocytes were then pelleted through 37% percoll, and red blood cells were lysed with ACK buffer.

### Ethics statement

The Institutional Animal Care and Use Committee of the National Institute of Allergy and Infectious Diseases approved all animal studies (#A4149-01). Studies were performed in accordance with recommendation of the Guide for the Care and Use of Laboratory Animals of the National Institutes of Health.

### In vitro restimulations and flow cytometry

For intracellular cytokine staining, isolated lung leukocytes were stimulated with soluble αCD3 and αCD28 (both at 1 μg/ml) in the presence of brefeldin A and monensin for 5 hours. Lung cells were stained with various combinations of the following mAb clones: αTNF MP6-XT22, αCD8 53.67, αIL-17A TC11-18H10.1, αCD4 RM4-5, αCD45.2 104, αIL-5 TRFK5, αCD44 IM7, αIFNγ XMG1.2, αIL13 eBio13A, αFoxp3 FJK-16S, αLy6G 1A8, αCD11b M1/70, αCD11c N418, αCD103 2E7, αCD68 FA11, αLy6C HK1.4, αSiglec F E50-2440, αT-bet 4B10, αPD-1 29F.1A12, αRORγt B2D, and αGATA-3 TWAJ. All FACS antibodies were purchased from eBioscience (San Diego, CA) and Biolegend (San Diego, CA). Analysis of CD4 T cell cytokine polyfunctionality was performed using SPICE version 5.3, downloaded from http://exon.niaid.nih.gov [72].

### Cytokine measurements

Lungs were homogenized in 1 ml PBS. The samples were then centrifuged at 16,000 g for 30 minutes at 4°C, and IFNα/β, IFNγ levels were determined in the supernatants using ELISA (PBL InterferonSource and eBioscience, respectively) according to the manufacturer’s recommendation. In some experiments, bronchoalveolar lavage fluid (BAL) was collected by lungs’ perfusion with 1 ml of cold PBS after partial tracheal resection using 22-gauge catheters. Mouse Cytokine Group I (23-Plex) was measured to quantify cytokine and chemokine concentrations in the supernatants obtained from mice, as described above, using Bio-Plex assay (Bio-Rad Laboratories, Inc., USA) following the manufacturer's instructions.

### Statistical analysis

Survival data from the animal experiments were analyzed using a two-group Wilcoxon test (GraphPad Prism analysis software). An unpaired t test was used for evaluation of the CFU in tissue burden studies. Two-group comparisons were done with the Student’s t test. When *P* values of <0.05 were obtained, differences were considered statistically significant.

## Supporting Information

S1 Fig Histopathology analysis, at day 28 post infection, of lungs from WT (upper panel), and *Ifnabr* ^*-/-*^ (lower panel) mice infected with 5 x 10^3^ CFU of *C*. *neoformans* strain H99; x200 magnification, hematoxylin and eosin stain. Data are representative of 3 independent experiments with n = 3 mice/group. Scale bar = 50 μm.
(TIF)

S2 Fig Treatment with recombinant IFNβ does not protect mice against *C*. *neoformans* infection. WT mice were intrapharyngeally infected with 5000 CFU of *C*. *neoformans* H99 and treated with rIFNβ twice weekly starting on the day of infection or left untreated and monitored for survival. Data are representative of 2 independent experiments with n = 5 mice/group.
(TIF)

S3 Fig Role of IFNaR in the production of chemokines and IFNγ in pICLC treatment of *C*. *neoformans* infection. BAL fluid was harvested from mice 7 days post-infection and KC (A), G-CSF (B), and CCL2 (C) were measured my multiplex analysis and IFNγ (D) was measured by ELISA. Data are representative of 3 mice/group and two independent experiments. Error bars represent the standard deviation (*, *P* < 0.05).
(TIF)

S4 Fig PICLC administration induces changes in the percentage of myeloid cells within the lung parenchyma. Mice were intrapharyngeally infected with 5000 CFU of *C*. *neoformans* H99. Lungs were harvested at day 20 post-infection and the percentage of each myeloid cell type that is negative for the intravascular stain was quantified. Data are representative of two independent experiments with n = 4–5 mice/group.
(EPS)

S5 Fig PICLC administration causes an influx of activated CD4's into the lung parnechyma. Mice were intrapharyngeally infected with 5000 CFU of *C*. *neoformans* H99 and lungs were harvested at day 20 post-infection. Data shows effector/memory CD4's that are negative for the intravascular stain. Data are pooled from 2 separate experiments with n = 5 mice/group.
(TIF)

S6 Fig Treatment with pICLC does not protect mice infected intravenously with *C*. *neoformans* H99. Mice were intravenously infected (via lateral tail vein injection) with 5000 CFU of *C*. *neoformans* H99 and treated with pICLC twice weekly starting on the day of infection or left untreated and monitored for survival. Data are representative of 2 independent experiments with n = 5 mice/group.
(TIF)
