# Supplementary figures and images for: Type I IFN Induction via Poly-ICLC Protects Mice against Cryptococcosis
Source: PLoS Pathog. 2015 Aug 7;11(8):e1005040. doi: 10.1371/journal.ppat.1005040 (PMC4529209; doi:10.1371/journal.ppat.1005040)

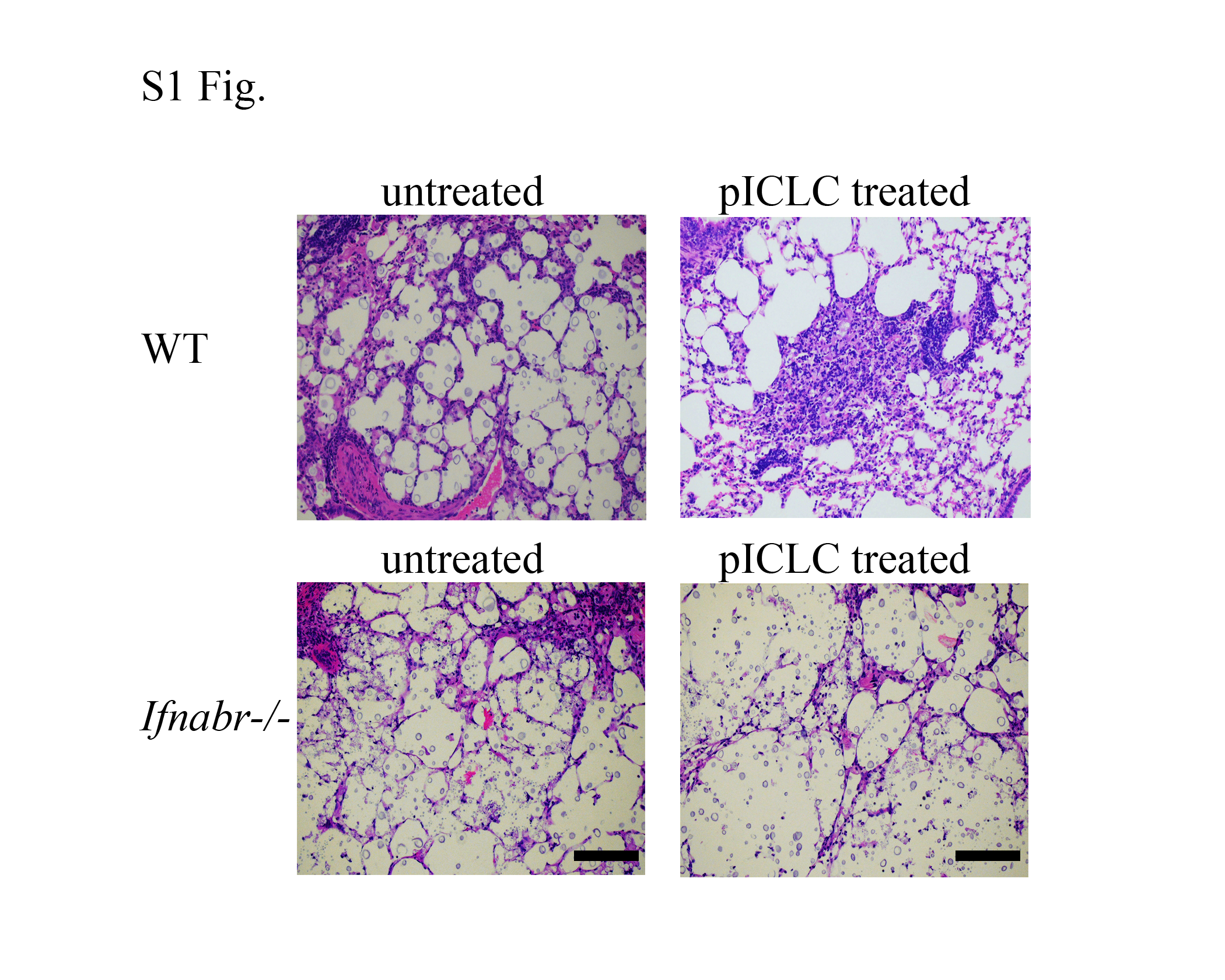

Supplement: S1 Fig — Data are representative of 3 independent experiments with n = 3 mice/group. Scale bar = 50 μm. (TIF) [file ppat.1005040.s001.tif]

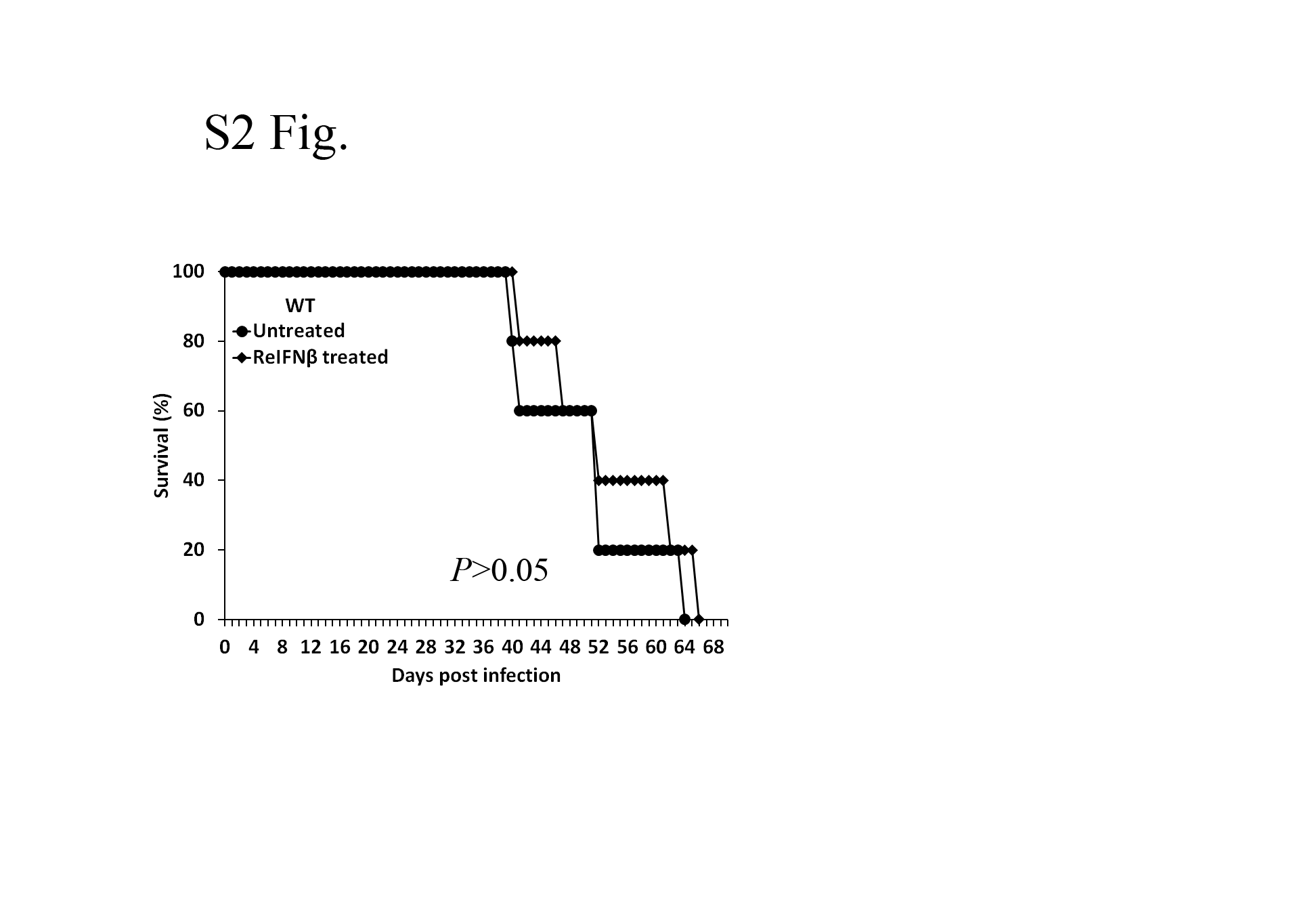

Supplement: S2 Fig — WT mice were intrapharyngeally infected with 5000 CFU of C. neoformans H99 and treated with rIFNβ twice weekly starting on the day of infection or left untreated and monitored for survival. Data are representative of 2 independent experiments with n = 5 mice/group. (TIF) [file ppat.1005040.s002.tif]

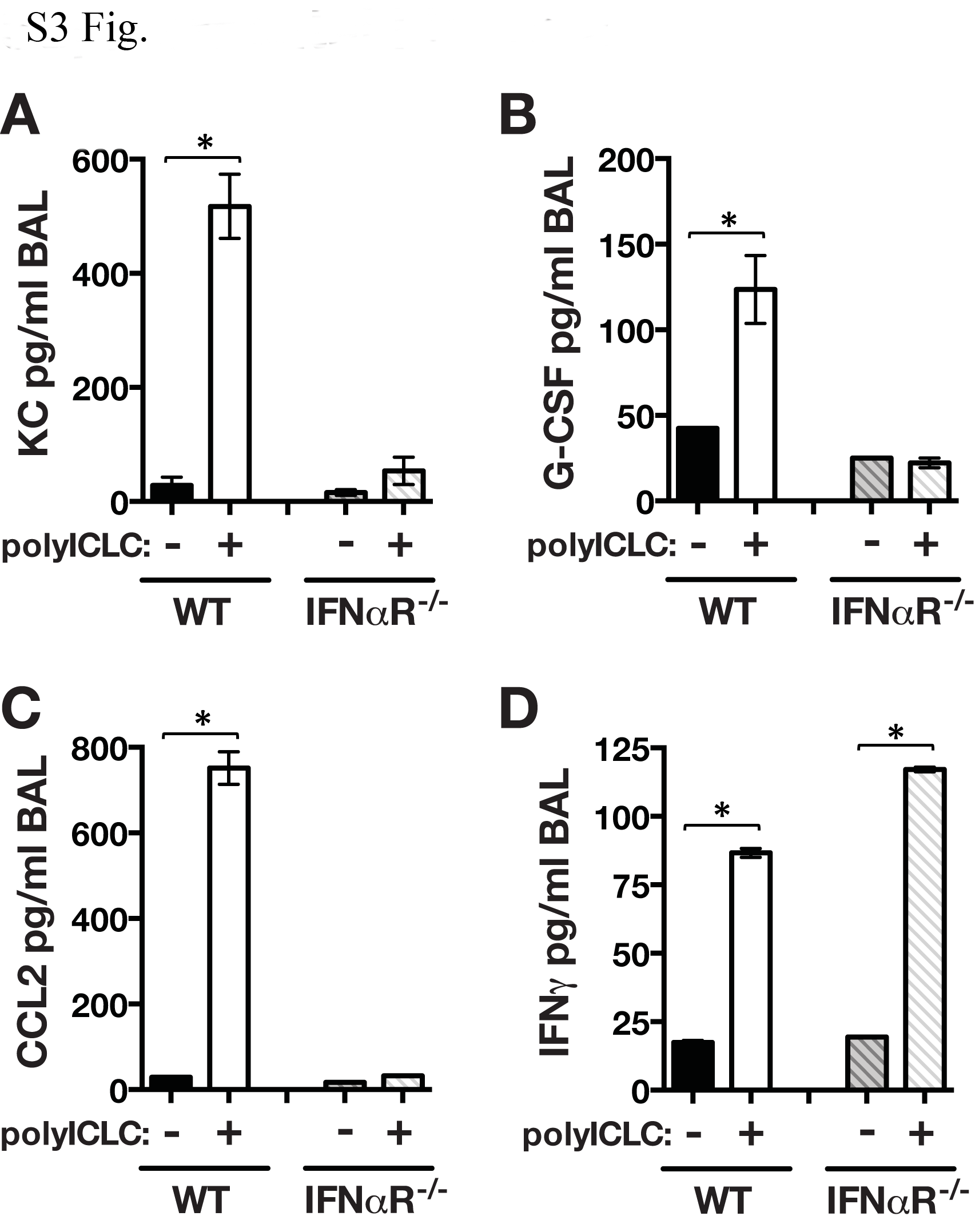

Supplement: S3 Fig — BAL fluid was harvested from mice 7 days post-infection and KC (A), G-CSF (B), and CCL2 (C) were measured my multiplex analysis and IFNγ (D) was measured by ELISA. Data are representative of 3 mice/group and two independent experiments. Error bars represent the standard deviation (*, P < 0.05). (TIF) [file ppat.1005040.s003.tif]

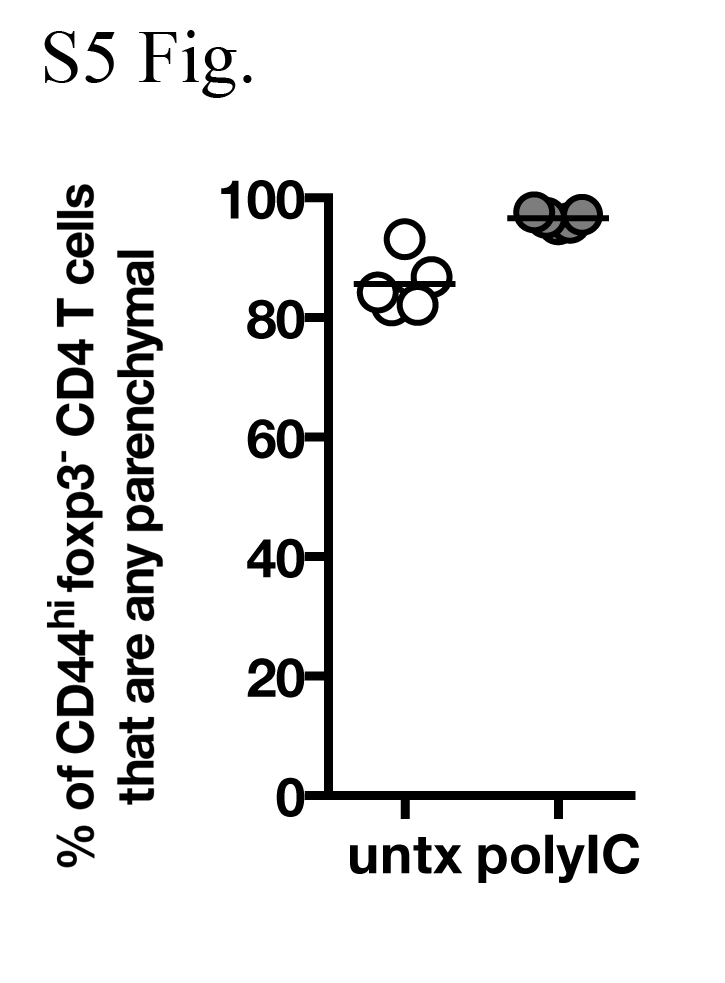

Supplement: S5 Fig — Mice were intrapharyngeally infected with 5000 CFU of C. neoformans H99 and lungs were harvested at day 20 post-infection. Data shows effector/memory CD4's that are negative for the intravascular stain. Data are pooled from 2 separate experiments with n = 5 mice/group. (TIF) [file ppat.1005040.s005.tif]

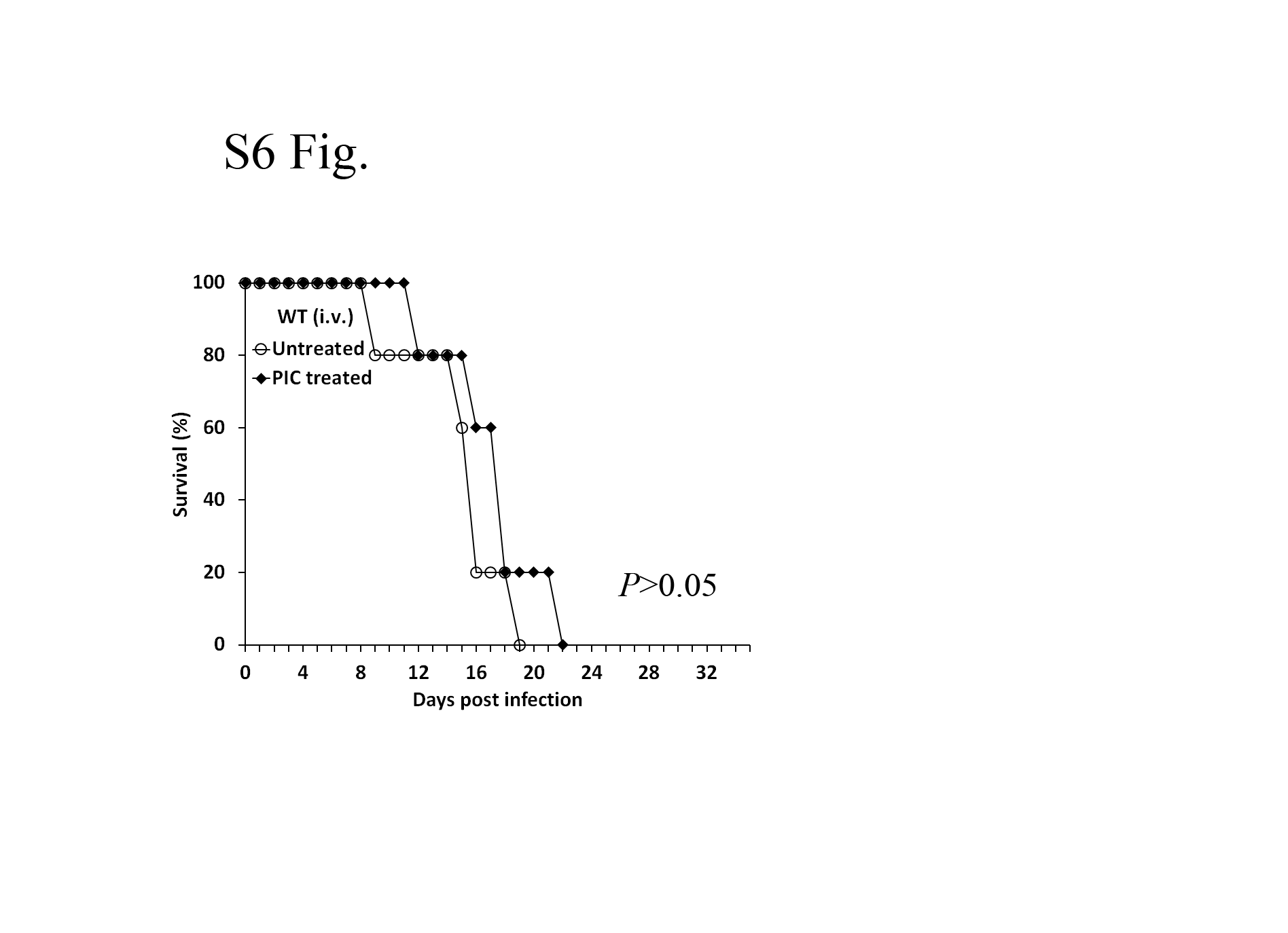

Supplement: S6 Fig — Mice were intravenously infected (via lateral tail vein injection) with 5000 CFU of C. neoformans H99 and treated with pICLC twice weekly starting on the day of infection or left untreated and monitored for survival. Data are representative of 2 independent experiments with n = 5 mice/group. (TIF) [file ppat.1005040.s006.tif]
